# Accurate de novo peptide sequencing using fully convolutional neural networks

**DOI:** 10.1038/s41467-023-43010-x

**Published:** 2023-12-02

**Authors:** Kaiyuan Liu, Yuzhen Ye, Sujun Li, Haixu Tang

**Affiliations:** 1 Luddy School of Informatics, Computing, and Engineering, Indiana University, Bloomington, 47408 IN USA; 2 Dengding BioAI Co., Ltd., Bloomington, USA

**Keywords:** Machine learning, Computational biology and bioinformatics, Proteomic analysis, Proteomics

## Abstract

De novo peptide sequencing, which does not rely on a comprehensive target sequence database, provides us with a way to identify novel peptides from tandem mass spectra. However, current de novo sequencing algorithms suffer from low accuracy and coverage, which hinders their application in proteomics. In this paper, we present *PepNet*, a fully convolutional neural network for high accuracy de novo peptide sequencing. PepNet takes an MS/MS spectrum (represented as a high-dimensional vector) as input, and outputs the optimal peptide sequence along with its confidence score. The PepNet model is trained using a total of 3 million high-energy collisional dissociation MS/MS spectra from multiple human peptide spectral libraries. Evaluation results show that PepNet significantly outperforms current best-performing de novo sequencing algorithms (e.g. PointNovo and DeepNovo) in both peptide-level accuracy and positional-level accuracy. PepNet can sequence a large fraction of spectra that were not identified by database search engines, and thus could be used as a complementary tool to database search engines for peptide identification in proteomics. In addition, PepNet runs around 3x and 7x faster than PointNovo and DeepNovo on GPUs, respectively, thus being more suitable for the analysis of large-scale proteomics data.

## Abstract (web summary)

De novo peptide sequencing allows the identification of peptides without requiring target databases. Here, the authors present PepNet, a convolutional neural network model for accurate de novo peptide sequencing that is capable of analysing large-scale proteomics data.

## Introduction

The past decade has witnessed great advances in mass spectrometry techniques, particularly liquid chromatography coupled tandem mass spectrometry (LC-MS/MS). With enhanced throughput and sensitivity, LC-MS/MS has become one of the most widely used approaches to functional studies of proteins at the whole proteome scale across various physiological (e.g., diseases) conditions in higher organisms including humans.

In a typical proteomics experiment, after MS/MS spectra are acquired, the first and arguably most important step is to identify the peptides from these spectra. Numerous algorithms have been developed to address this problem, which mostly fall into three categories: protein database searching, spectral library searching, and de novo sequencing. Protein database searching is the predominant approach used for peptide identification. The peptide sequence tag method^1^ and the Sequest algorithm^2^ were the early algorithms of this category. More recent developments include Mascot^3^, X!Tandem^4^, OMSSA^5^, MyriMatch^6^, Protein Prospector^7,8^ and MSGF+^9^. Those methods compare the experimental spectra with the theoretical spectra generated from the peptides in a protein database and report those likely true peptide-spectrum matches (PSMs).

By contrast, the spectral library search approach compares newly acquired MS/MS spectra against a library containing previously characterized experimental spectra that were used in early computational analysis^10,11^. Thanks to the improved repeatability and reproducibility of MS/MS data as well as the increasing availability of massive experimental spectra (e.g., from the proteomics data repository^12^ and large-scale synthetic peptides projects^13^), the spectral library search approach has become more increasingly adopted and is implemented in software tools such as X!hunter^14^, SpectraST^15^ and msSLASH^16^.

Finally, de novo sequencing algorithms attempt to derive a peptide sequence directly from its MS/MS spectrum without using references such as a spectral library or a protein sequence database^17^. Many de novo sequencing algorithms adopted a graph theoretical formulation to compute the longest path in the spectrum graph by employing a dynamic programming algorithm^18,19^ and adaptive scoring schemes^20–22^. With the advancement of high-resolution MS instruments, the performance of de novo sequencing algorithms improves significantly^23,24^, in particular with more sophisticated scoring schemes. More recently, DeepNovo^25–27^ and its successor PointNovo^28^ were developed using deep learning algorithms, which automatically learn the fragment ion patterns relevant to peptide sequences from massive MS/MS spectra of peptides and reported improved performance. These methods exploited a deep neural network (DNN) architecture to capture the dependence among fragment ions in the input tandem mass spectra that were subsequently used to construct the peptide in a sequential order. Although these methods have exhibited better performance than conventional de novo sequencing algorithms, we observed they can sequence relatively fewer long peptides, in particular from charge 3+ MS/MS spectra, perhaps due to the challenge of modeling complex long-range patterns among fragment ions. On the other hand, the convolutional neural network (CNN) architecture adopted by PredFull^29^ for full MS/MS spectra prediction demonstrated the advantage of CNN to learn complex patterns in MS/MS spectra.

In this work, we develop a deep learning model called PepNet that achieves substantially improved performance for de novo peptide sequencing from tandem mass spectra compared to previous methods. PepNet demonstrates strong performance on MS/MS spectra data from both human and various non-human organisms. On average, PepNet can sequence 2.5-19x more unidentified spectra than other tools at comparable levels of precision. These results suggest that PepNet significantly advances the accuracy of de novo peptide sequencing, and thus could serve as a complementary tool to database search engines for peptide identification in proteomics.

## Results

### Accurate HCD-MS/MS spectra de novo sequencing by deep learning

**Fig. 1:**
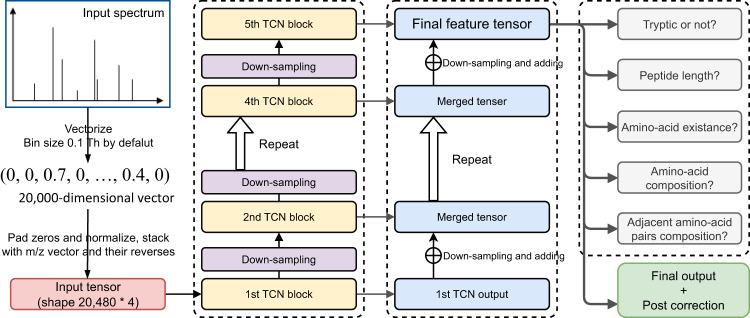
The Neural Network Architecture of PepNet. The PepNet Network uses a series of temporal convolutional network (TCN) and down-sampling layers to encode the input MS/MS spectrum, from which the global and local information in the spectrum is fused into a single feature tensor and then decoded into the peptide sequence.

We present a deep learning algorithm, *PepNet*, that directly outputs the peptide sequence from a given HCD-MS/MS spectra with high accuracy. As depicted in Fig. 1, the input for our model is a 20,480 × 4 matrix that represents the input spectrum (for details see the *Method* section). The input matrix will go through five continuing temporal convolutional network (TCN) blocks^30^ and down-sampling layers to capture the relationships between observed peaks, as depicted in the TCN branch of Fig. 1. These five TCN blocks work on different resolution levels, capturing global and local information of the spectra, and a merging branch (bottom-up branches in Fig. 1) is used to fuse the global and local information into a single feature tensor. The feature tensor is then converted by a softmax decoding layer into a probability matrix of size 32 × 23, in which each column contains the softmax probabilities of the target amino acid at the corresponding position in the peptide (as illustrated in Supplementary Fig. S1). Here, the 32 columns in the probability matrix represent the up to 30 positions in the target peptide (which of a maximum length of 30), one ending character, and one or more padding characters. The 23 rows represent the one-hot probability of the following characters: 20 characters of standard amino acids (at that position), one starting character (reserved for compatibility but not currently used), one ending character, and one padding character. From the final probabilities matrix, we derive the optimal peptide sequence by choosing the amino acid with the highest probability at each position. Additionally, the feature tensor is also sent to several relatively easy auxiliary tasks (auxiliary tasks branch in Fig. 1) to guide and regularize the target de novo sequencing task.

It is worth noting that, we do not force the model to output a peptide that has the matched precursor mass; instead, we design the loss function to encourage the model to output the peptide sequence containing as many correct amino acids as possible. This approach can prevent the model from outputting a peptide that has the desirable amino acid composition, but neglecting the sequence information in the input MS/MS spectrum, in particular during the early stage of the training process when the model has not learned sufficient knowledge.

We collected the HCD spectra from multiple peptide spectral libraries to build our learning dataset, which is split into three sets of training, validation, and testing. Here we retained spectra with charges of 1+ to 8+ and from peptides of length no longer than 30, which resulted in 3,041,570 HCD-MS/MS spectra from 1,066,296 distinct peptides (see the *Method* section for details). We then employed two independent proteomics datasets to evaluate the performance of PepNet, in which we excluded all spectra that shared the same peptides as our learning dataset. We implemented PepNet using Tensorflow^31^ and trained it using the RAdam optimizer^32^ for 50 epochs (using a learning rate of 0.02). The entire PepNet model contains about 77 million parameters. Notably, we did not distinguish spectra that were acquired by different types of instruments during training and testing, as we observed that the HCD spectra acquired using different instruments (e.g., Orbitrap, Fusion, or Q-Exactive) could all be sequenced with high accuracy using a single model. PepNet is released as open-source software at GitHub (https://github.com/lkytal/PepNet). It is also available online at https://denovo.predfull.com/.

### Evaluation criteria

We evaluated the performance of PepNet over two proteomics datasets. For these datasets, we re-used the database search results from the original publication (which was conducted using MaxQuant^33^), and only preserved results with a false discovery rate (FDR) no greater than 1% and with a precursor mass difference no greater than 10 ppm as the ground truth. Then we computed two measurements to evaluate the accuracy of the de novo peptide sequencing: the positional accuracy, defined as the fraction of correct amino acid residues reported by de novo sequencing algorithms, and the peptide-level accuracy, defined as the fraction of spectra that the sequenced peptides are completely correct. Note that for both measurements, we do not distinguish the amino acids of Leucine (Leu) and Isoleucine (Ile). These two accuracy measurements, as well as the Precision-Coverage curves of PepNet, were compared with the current state-of-the-art de novo peptide sequencing algorithm PointNovo^26^ and its predecessor DeepNovo^25^. Finally, we present here only the de novo sequencing of 2+ and 3+ HCD spectra of unmodified peptides as they are most common in shotgun proteomics data, although this model can sequence spectra of other charges (charge 1+ to 8+), and could be easily extended to sequencing spectra from the peptides containing common post-translational modifications (PTMs).

In the following sections, we report the performance of PepNet in comparison with DeepNovo and PointNovo on a large-scale human proteomics dataset and a large-scale dataset acquired from multiple non-human organisms. We first compare their accuracy on the subset of spectra identified by MaxQuant in these datasets to show that PepNet achieves higher sequencing accuracy, and then show that PepNet can sequence more peptides than the other two tools (under the cutoffs corresponding to the same precision level) on those MS/MS spectra that were not identified by MaxQuant. All evaluations were conducted on an 8x NVIDIA A6000 server. Notably, when executed on a single NVIDIA A6000 GPU, PepNet can sequence 10,000 spectra in about 59 seconds, which is around 3 times faster than PointNovo (about 161 seconds) and over 7 times faster than DeepNovo (about 431 seconds).

### Performance evaluation on a large-scale human proteomics data set

We first evaluated the performance of PepNet using the HCD spectra from a large-scale human proteomics project (ProteomExchange ID: PXD019483) in comparison with PointNovo and DeepNovo. In this section, we report the de novo sequencing results on the subset of identified spectra from the original study^34^, while the de novo sequencing results on the remaining unidentified spectra are reported in a separate section below. After filtering, 393,206 charge 2+ and 206,930 charge 3+ spectra identified by MaxQuant were preserved, adhering to the criteria of FDR ≤ 1% and precursor mass difference ≤ 10 ppm, as previously described. Note that we only use spectra of peptides not present in the training dataset for testing, for a fair evaluation.

**Fig. 2:**
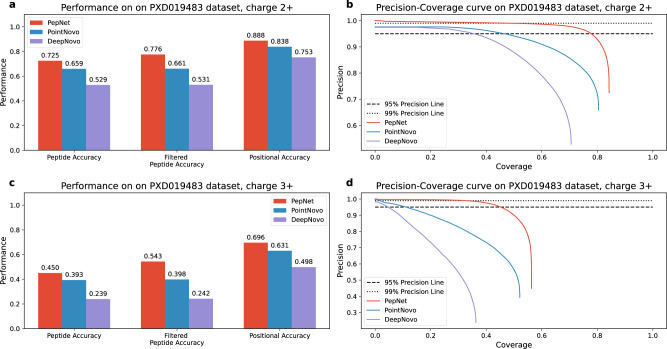
The accuracy and the Precision-Coverage curves of PepNet, PointNovo, and DeepNovo on the charge 2+ (upper half) and charge 3+ (lower half) spectra in the human proteomics dataset. Here, the “Filtered Peptide Accuracy" is referred to as the peptide-level accuracy on the sequenced peptides after removing the sequencing results with unmatched precursor masses (i.e., over 10 ppm). The dotted lines represent the precision levels of 0.95 and 0.99, respectively. **a** Accuracy on charge 2+ spectra, **b** Precision-Coverage curves on charge 2+ spectra, **c** Accuracy on charge 3+ spectra, **d** Precision-Coverage curves on charge 3+ spectra.

As shown in Fig. 2, PepNet gave significantly more accurate peptide sequencing than PointNovo and DeepNovo. PepNet achieved peptide-level accuracy of 0.725 (i.e., 72.5% of identified peptides are completely correct) and 0.450 for charge 2+ and 3+ spectra, respectively. And the peptide accuracy increased to 0.776 and 0.543, respectively, after filtering out the apparently wrong identifications with unmatched precursor masses. On positional accuracy, PepNet achieved 0.888 and 0.696 on charge 2+ and 3+ spectra, respectively. All these results are substantially superior to those of PointNovo and DeepNovo.

Also, the Precision-Coverage curves depicted in Fig. 2 demonstrate that PepNet can sequence more peptides with higher accuracy. Here, for each point in a Precision-Coverage curve, the Coverage (represented in the x-axis) is computed as the fraction of ground truth peptides covered by the sequencing results that with quality scores above the specific threshold, while the Precision is computed as the fraction of correctly sequenced spectra (i.e., the sequenced peptides matching the peptides identified by MaxQuant) among all spectra sequenced by the respective algorithm with the quality scores above the same threshold. When applying a precision threshold of 95%, PepNet exhibits superior performance by sequencing a much larger fraction of spectra as compared to PointNovo and DeepNovo. Remarkably, when the precision threshold is elevated to 99%, PepNet can still sequence approximately 50% and 32% of peptides for charge 2+ and 3+ spectra, respectively, while neither PointNovo nor DeepNovo can reach this level of precision, resulting in no remaining results.

**Fig. 3:**
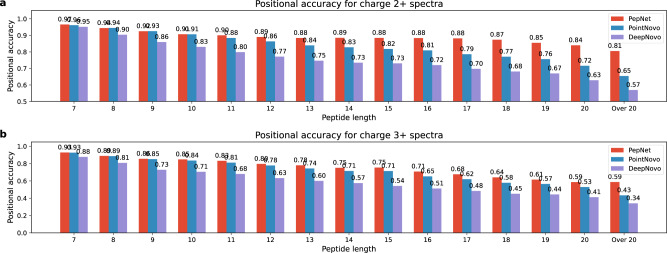
Impact of peptide length on sequencing accuracy. The positional accuracy of PepNet, PointNovo, and DeepNovo on peptides of different lengths for the spectra of charge 2+ (**a**) and charge 3+ (**b**) in the human proteomics dataset.

We further investigated the performance of PepNet, PointNovo, and DeepNovo on spectra from the peptides of different lengths, as depicted in Fig. 3. Not surprisingly, the positional accuracy of PepNet (as well as PointNovo and DeepNovo) reduced with the increasing lengths of peptides, perhaps because 1) longer peptides are more challenging to be de novo sequenced due to more complex fragmentation patterns, and 2) the training dataset contains relatively fewer training samples of longer peptides. Nevertheless, the performance of PepNet is consistently better than PointNovo and DeepNovo, especially for longer peptides.

### Performance evaluation on proteomics data from non-human organisms

Next, we evaluated the performance of PepNet on the proteomics data collected from a variety of non-human organisms. We used the HCD spectra from a large-scale proteomics project (ProteomExchange ID: PXD014877) aiming to elucidate the evolutionary landscape of the proteomes in 56 organisms including bacteria, fungi, plants, and animals. The numbers of testing spectra of each organism on the dataset PXD014877 can be found in Supplementary Fig. S2. Similar to the results shown above, here we report the de novo sequencing results on the subset of identified spectra reported by the original study^34^ (filtered by the criteria of FDR ≤ 1% and precursor mass difference ≤ 10 ppm).

**Fig. 4:**
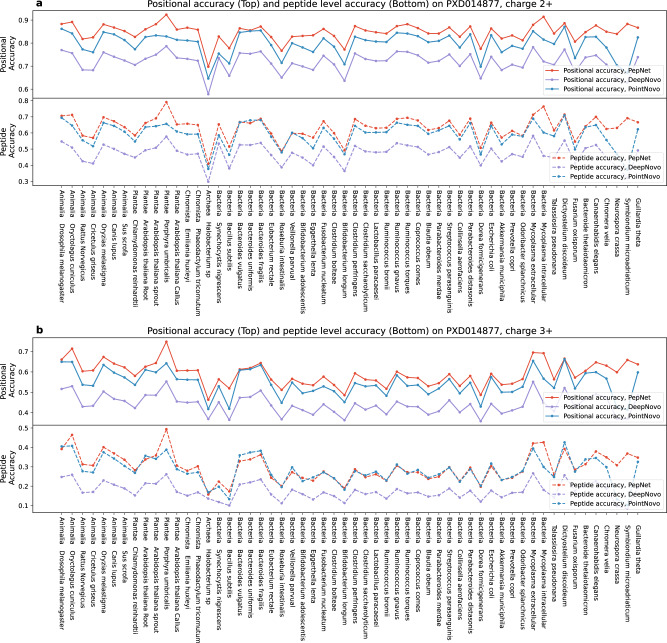
Sequencing accuracies of peptides from non-human organisms. Performance of PepNet, PointNovo, and DeepNovo are shown on the spectra of charge 2+ (**a**) and charge 3+ (**b**) in the proteomics datasets acquired from different non-human organisms.

As illustrated in Fig. 4, the performance of PepNet remains consistent across various organisms, and is consistently higher than PointNovo and DeepNovo in terms of positional accuracy. Notably, the performance of PepNet on the proteomics data from these varieties of organisms is comparable with the performance on the human proteome data (mentioned in the above section), indicating that even though PepNet was trained using MS/MS spectra mostly from human peptides, the model is well-generalized for the de novo sequencing of non-human peptides.

To further assess PepNet’s ability for sequencing peptides from no-human organisms, we arbitrarily selected two organisms ("Sus scrofa" and “Arabidopsis thaliana Callus", respectively), and investigated if the peptides sequenced by PepNet from the proteomics data match the proteins from the respective organism. Specifically, among the peptides sequenced by PepNet on all spectra (including both identified and unidentified spectra by MaxQuant) from each of these two organisms with the score above the cutoff corresponding to the 95% peptide-level accuracy, we observed a substantial fraction (58.2% and 47.2%, respectively) matched the peptides (with at most one mutation) from the corresponding organisms (see Supplementary section “Sequencing results on Selected Organisms" for details). This result again confirms that PepNet can be used to sequence non-human peptides.

### De novo sequencing of spectra not identified by database search engines

In this section, we demonstrate that PepNet can identify a large fraction of MS/MS spectra that cannot be confidently identified by the database search engines. We applied PepNet to the subset of MS/MS spectra that were not identified by MaxQuant in the human proteomics dataset (ProteomExchange ID: PXD019483, as described above). We then computed a Quality Score for each sequenced peptide as the product of the probabilities of all amino acids in the sequenced peptide.

**Fig. 5:**
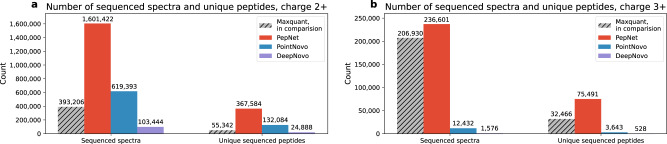
Numbers of sequenced spectra and peptides. The numbers of de novo sequenced spectra and unique peptides by PepNet are shown in comparison with those by PointNovo and DeepNovo on spectra of (**a**) charge 2+, and (**b**) charge 3+, respectively.

As PepNet, PointNovo, and DeepNovo all output an estimated quality score for each sequencing result, we need to determine the appropriate cutoff for each model. For a fair comparison, we choose cutoffs for PepNet, DeepNovo, and PointNovo that allow each algorithm to yield > = 95% full peptide accuracy on the *identified spectra*. After removing sequenced peptides with unmatched precursor masses or with a quality score lower than the selected cutoffs, we observed that PepNet sequenced much more spectra (Fig. 5). On the charge 2+ spectra, PepNet sequenced 2.5x more spectra than PointNovo and 15x more spectra than DeepNovo. The difference is even greater on charge 3+ spectra, on which PepNet sequenced about 19x more spectra than PointNovo, while DeepNovo sequenced almost no spectra under the expected accuracy. While comparing the unique peptides sequenced from these spectra, PepNet sequenced even greater folds of unique peptides than PointNovo and DeepNovo (Fig. 5), suggesting its superior performance for sequencing the spectra that are not identified by database search engines. Notably, the gap of performance between PepNet and PointNovo/DeepNovo is much greater on the charge 3+ spectra, which suggests that PointNovo and DeepNovo are trained with the bias toward the 2+ spectra, as the 2+ spectra are much more abundant in the training dataset. On the other hand, the Multitask Learning (MTL) strategy adopted by PepNet may contribute to its performance on charge 3+ spectra.

**Fig. 6:**
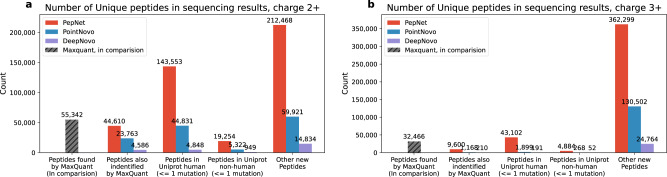
Peptides sequenced on unidentified spectra. The number of unique peptides sequenced by PepNet are comared with those by PointNovo and DeepNovo on the unidentified spectra and their matches with the proteins in Uniprot (identical or with one mutation) for spectra of charge 2+ (**a**) and charge 3+ (**b**).

To understand what kind of unidentified spectra can be sequenced by PepNet, we search these sequenced peptides against the UniProt human proteins database^35^ (20,383 human proteins) using RAPSearch2^36^ (also, without distinguishing amino acids of Leu and Ile). We observed that for charge 2+ spectra, only 44,831 and 4,848 unique peptides sequenced by PointNovo and DeepNovo matched with human proteins in Uniprot with at most one mutation (Fig. 6 and Supplementary Fig. S4), respectively, which are only around 31.3% and 3.3% compared to those discovered by PepNet (143,553 unique peptides). This is consistent with their Precision-Coverage curves (Fig. 2) on the spectra identified by MaxQuant, as presented in the previous section. Similar trends were observed in other comparative results among PepNet, PointNovo and DeepNovo, such as the number of sequenced unique peptides matching with peptides identified by MaxQuant, the number of unique peptides matching with non-human proteins in Uniprot, and the number of unique new peptides. For the charge 3+ spectra, the unique peptides sequenced by PointNovo and DeepNovo are nearly negligible. Finally and not surprisingly, most of the peptides sequenced by PointNovo and DeepNovo that match with human proteins in Uniprot were also sequenced by PepNet (see Supplementary Fig. S5).

**Fig. 7:**
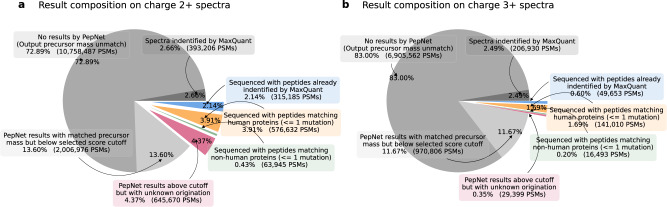
The composition of the sequencing results. The composition of the sequenced peptides on the spectra of  charge 2+ and charge 3+ are shown in (**a**) and (**b**), respectively. Here, the pull-out parts represent sequenced spectra with a matched precursor mass (≤10 ppm) and a quality score ≥ 95% precision cutoff.

We further search PepNet’s sequencing results against the Uniprot proteins database^35^ (568,002 proteins, including 20,383 human proteins). As shown in Fig. 7, for charge 2+ spectra, 315,192 spectra were sequenced with peptides that were also identified by MaxQuant. These spectra are likely to have relatively lower quality and thus were not identified by MaxQuant due to the FDR cutoff. Meanwhile, PepNet sequenced 576,603 spectra (1.47x, compared to the number of spectra identified by MaxQuant) as the peptides that either matched perfectly with human proteins or with one mutation (substitution, insertion, and deletion). Besides, another 63,759 spectra were sequenced as peptides that match non-human proteins in Uniprot with at most one mutation. While for charge 3+ spectra, the number of spectra sequenced by PepNet is also comparable to the number obtained by MaxQuant. These results suggest that the de novo sequencing results produced by PepNet are complementary to the database search engines (such as MaxQuant). Further analyses of the de novo sequenced peptides that were not identified by database search engines may lead to the discovery of additional peptides/proteins expressed in the proteome samples, including those absent from the target protein database. For instance, sequencing results that were illustrated in pink in Fig. 7, represent the sequenced spectra with high-quality scores and matched precursor masses but do not match any sequence in the proteins database.

Besides the large advantage in the number of sequenced spectra and peptides by PepNet, we observed that the average length of the peptides sequenced by PepNet is notably longer than the average length of peptides sequenced by PointNovo and DeepNovo (Supplementary Fig. S3), suggesting that PepNet is capable of sequencing longer (and thus more complex) peptides, which is consistent with the observation on the sequencing results of the spectra identified by MaxQuant (see Fig. 3).

**Fig. 8:**
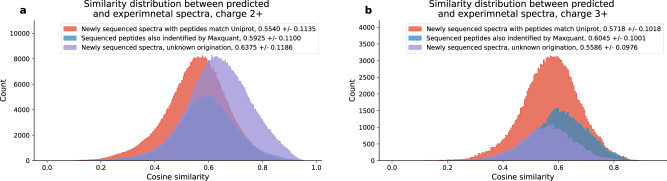
The similarity between the experimental and predicted spectra of sequenced peptides. The distributions of the similarities between the experimental and predicted spectra (by PredFull) on the sequenced peptides of charge 2+ and  charge 3+ are shown in (**a**) and (**b**), respectively.

Finally, we expect the spectra sequenced by PepNet with high quality scores (above 95% precision) and matched precursor masses but not matching with any proteins in Uniprot (i.e., illustrated in pink in Fig. 7) should contain a considerable number of correctly sequenced peptides. Because they do not match with any proteins in Uniprot, we employed two orthogonal measurements to validate these results. Firstly, we investigated the cosine similarity between the experimental spectra and the “theoretical spectra" predicted by Predfull^29^ using the sequenced peptides. We observed that the distribution of spectra similarities for these newly sequenced peptides is very similar to that of the peptides matching proteins in Uniprot, with both average similarities around 0.6 (Fig. 8). Moreover, these average similarities are not far from the average cosine similarities between replicates of spectra identified by MaxQuant (Supplementary Fig. S7). Secondly, we compared the retention time of the experimental spectra and the retention time of sequenced peptides predicted by DeepLC^37^, and observed that they are quite similar (Supplementary Fig. S8). Together, these results show that even though these peptides are not highly similar to any protein in Uniprot, the quality of these sequencing results is still comparable with those with Uniprot matches, implicating many of these sequencing results are likely to be correct. Future work will focus on further validating these results and exploring the potential applications of PepNet in a wide range of biological and clinical studies.

### Performance of PepNet on proteomics data from Data-Independent Acquisition (DIA)

We further demonstrate that PepNet is also capable of the de novo sequencing of the MS/MS spectra derived from the data acquired by using Data Independent Acquisition (DIA). We compared the performance of PepNet against PointNovo and DeepNovo-DIA on a dataset acquired from a human plasma sample (provided by DeepNovo-DIA ^27^ in their original publication), which contains a total of 56,879 MS/MS spectra derived from DIA data. Notably, DeepNovo-DIA is a refined DeepNovo model using DIA-derived MS/MS spectra as training data; by contrast, we directly applied the PepNet model trained to the HCD-MS/MS spectra from Data Dependent Acquisition (DDA) as described above without any further refinement.

**Fig. 9:**
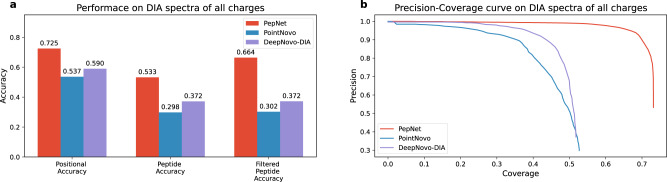
Peptide sequencing accuracies on DIA spectra. The sequencing accuracy (**a**) and the Precision-Coverage curve (**b**) of PepNet, PointNovo, and DeepNovo-DIA are compared on a dataset of DIA-derived MS/MS spectra. The Filtered Peptide Accuracy is referred to as the peptide-level accuracy on the sequenced peptides after removing those with unmatched precursor masses (i.e., over 10 ppm).

The performance of PepNet is significantly better than PointNovo, which is not surprising. What is encouraging is that PepNet also outperforms DeepNovo-DIA, which was fine-tuned specifically for the DIA-derived spectra. As shown in Fig. 9, PepNet achieved a positional accuracy of 0.725 and peptide-level accuracy of 0.533 on the combined set of 2+ and 3+ spectra (the separated performance on charge 2+ and 3+ are shown in Supplementary Fig. S9). These results showed PepNet achieved comparable performance on the DIA-derived spectra as on the DDA-acquired spectra, indicating that PepNet is robust for de novo sequencing of not only the DDA-acquired MS/MS spectra but also the MS/MS spectra derived from DIA data.

### Observed factors related to performance

**Fig. 10:**
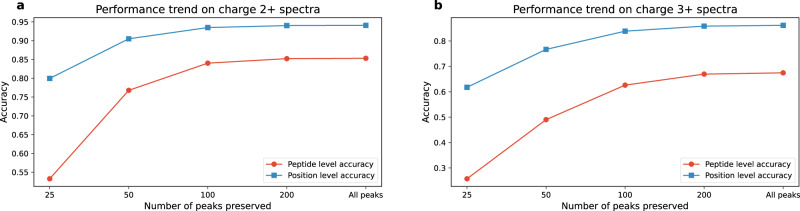
The impact of retained number of peaks on the performance of PepNet. The positional and peptide-level accuracy of PepNet are shown on the input charge 2+ (**a**) and 3+ (**b**) spectra in the testing dataset, on which different numbers of most intensive peaks are retained.

We found multiple factors influencing the performance of PepNet that are worth discussing. We observed that the number of most intensive peaks retained in the input MS/MS spectrum to PepNet has a significant impact on its performance, as shown in Fig. 10. The performance of PepNet significantly decreases when fewer peaks are retained in the spectra given as input to the PepNet. This result indicates that the peaks of low intensities, including some non-backbone fragment ions that were considered as “noise" peaks and thus ignored by many conventional de novo sequencing algorithms, also provide useful information for the PepNet model, especially for determining the residues at some positions where the supportive backbone ions are missing.

Besides, we observed that the positional accuracy by PepNet shared a similar trend for the sequenced peptides of different lengths. As shown in Supplementary Fig. S6, for the charge 2+ spectra, the positional accuracy is relatively low for the first few amino acid residues at the N-terminus, and then increases before decreasing gradually until the last residue at the C-terminus. It is not surprising that the C-terminal residue is determined accurately because most tested spectra are tryptic peptides. This trend of sequencing error distribution is largely due to the coverage of the observed fragment ions: the first few residues are easier to determine because the *b*_1_ and *b*_2_ (and even some *b*_3_) ions are often missing in HCD spectra, while y-ions are weaker than b-ions, causing the C-terminal residues are harder to be determined.

## Discussion

In this research, we present PepNet, a deep learning model designed for accurate de novo peptide sequencing from HCD-MS/MS spectra. We first demonstrate that PepNet is capable of sequencing human MS/MS spectra with high accuracy, and then we show that PepNet can perform consistently well across MS/MS data from many non-human organisms. Furthermore, the de novo sequencing results on unidentified spectra demonstrate that PepNet has the capability to discover numerous identifications from spectra that MaxQuant overlooks, yielding several times more identifications than those previously detected by MaxQuant. This suggests that although PepNet was trained using peptides sequenced by database searching tools like MaxQuant, PepNet (and other de novo algorithms) is not limited by the specific peptide knowledge of the training samples. Therefore, PepNet can be used as a powerful tool for proteomic data analysis, especially when a comprehensive target protein sequence database is not available (e.g., in metaproteomics^38^).

We believe that the ability to sequence peptides with high accuracy will enable the increasing applications of de novo peptide sequencing in proteomics data analysis. In addition to the peptide sequencing for HCD spectra as presented in this paper, PepNet can be extended to the MS/MS spectra acquired by using other fragmentation methods, such as the electron transfer dissociation (ETD), Electron-Transfer/Higher-Energy Collision Dissociation (EThcD), photodissociation (PD) and the infrared multiphoton dissociation (IRMPD). These methods were often considered to result in complex MS/MS spectra, in which those rich information embedded in the complex MS/MS spectra may hopefully improve the accuracy of de novo peptide sequencing. Therefore, we anticipate that PepNet will enhance the efficiency of proteomics data analysis and will benefit the life science research community.

## Methods

### Representation of the MS/MS spectra

We represent the input MS/MS spectrum as a one-dimensional (1-D) vector by binning the spectrum with a given bin width. We considered only the peaks within the range of mass-to-charge ratio (m/z) between 0 and 2000 as most experimental spectra do not contain peaks with m/z above 2000. By default, we use a bin width of 0.1 Th, which yields a vector representation of 20,000 dimensions. Based on our experiment, using an even smaller bin size (i.e., higher mass resolution) did not improve the performance of de novo sequencing but required longer running times. Finally, we removed the precursor peak in each spectrum, and normalized each spectrum by dividing each peak over the intensity of the maximum peak in that spectrum, similar to our previous work for MS/MS spectrum prediction^29^.

### Deep neural network architecture

The PepNet model is primarily based on the temporal convolutional network (TCN) blocks^30^. We expect PepNet to have a large enough receptive field to capture the potential associations between distant peaks in the MS/MS spectra while maintaining a relatively low computational cost. TCN blocks, which are stacked dilated convolutional layers with their dilation rate growing exponentially, can efficiently cover a large receptive field by stacking more dilated layers. The size of the effective receptive fields of each TCN block can be calculated by 1 + 2*(*kernel*_*size* − 1)*(2^*n*^ − 1)^30^, where *n* denotes the number of the dilated layers.

Firstly, zero-padding is applied to the end of the vector representation of each spectrum, extending its dimension from 20,000 to 20,480. This extension avoids non-integer dimensions in the following down-sampling layers. The m/z array is also extended into a 20,480 dimensional vector in the same manner. These two vectors, along with their reversed counterparts, are then stacked together to create an input matrix of a shape 20,480 × 4. Then, as depicted in the TCN branch of Fig. 1, five consecutive TCN blocks and down-sampling layers are designed to capture the relationships between observed peaks. These lower TCN blocks are designed to have larger receptive fields but have fewer channels, while after each down-sampling layer, the following TCN block will have halved receptive fields but with approximately 1.5 times more channels.

Although those TCN blocks are capable of extracting most of the information from the input MS/MS spectrum, they work at different levels of resolution, thereby obtaining complementary information: the topmost TCN block emphasizes the detailed local structures of the input spectrum whereas the bottom-most block considers mostly the global features of the spectrum. To fuse global and local information, we introduce a bottom-up branch that merges output from all TCN blocks into a single feature tensor, as depicted in Fig. 1.

After that, meta-information (e.g. charge of the spectra; M/z of the precursor; normalized collision energy, if known) will be transformed by a linear layer (with Sigmoid activation function) and concatenated to the end of the previously mentioned feature tensor to yield the final feature tensor, which is then converted into a final probabilities matrix of the size 32 × 23 by a softmax decoding layer. As each column in the matrix represents the probabilities of each amino acid at the corresponding position (20 characters of standard amino acids and 3 special symbols, as stated in the *Result* section), we can derive the optimal peptide sequence by choosing the amino acid with the highest probability in each column, until we meet the position at which the ending character is of the highest probability. As a post-processing step of this strategy, if the theoretical mass of the inferred peptide differs from the experimental precursor mass by more than 10 ppm, we attempt to check if any sub-optimal peptide has matched precursor mass: we substitute the amino acid at each position with the one with the second-highest probability; if the resulting peptide has a matched precursor mass, it will be the output as the finalde novo sequencing result; otherwise, the original optimal peptide sequence will still be reported.

In addition to the de novo sequencing task, several relatively easy tasks (the auxiliary task branch in Fig. 1) are trained simultaneously to achieve better performance, as described in the following section.

### Optimizing hyper-parameters for PepNet’s main architecture

When designing the primary architecture of PepNet, crucial hyperparameters such as the number of TCN blocks, kernel sizes, and the number of channels were determined based on the trade-off between the runtime and the performance. We employed five TCN blocks because more blocks show no improvement in performance, as the receptive field of five TCN blocks is already large enough, as shown in Supplementary Fig. S11. Regarding the number of convolutional channels, we observed that the performance of PepNet continues to improve as it increases, although the improvement gradually diminishes. We finally selected the number of layers as [192, 288, 384, 576, 768] for each TCN block, since more layers will be too computationally expensive. Also, Supplementary Fig. S11 shows the performance of PepNet under different kernel sizes of TCN layers: the performance hardly improves after the kernel size reaches 5*2^5^.

### Loss functions and auxiliary tasks

Well-designed loss functions are critical for achieving optimal training performance in deep neural networks. We employ the average cross-entropy on non-padding positions (termed masked_ce_loss) as the major part of the loss, while monitoring the average accuracy on non-padding positions to select the optimal model. As emphasized in the previous sections, we do not enforce the model to output a peptide sequence with an exactly matched precursor mass, which is, however, an important constraint for de novo peptide sequencing. Thus we add the mean absolute error (MAE) of the predicted sequence and the mass of the true sequence to the final loss functions, i.e., *loss* = masked_ce_loss+ *c* * MAE. Here, the weight *c* should be sufficiently small to prevent the MAE part from dominating the loss during the initial training stages, when the model has not learned enough knowledge to generate a reasonable sequence. In practice, we manually select *c* to ensure that c*MAE does not exceed 10% of the total loss.

Auxiliary tasks also play a crucial role in improving the model’s performance, including 1) whether the target peptide is a tryptic peptide; 2) the length of the target peptide; 3) the existence of amino acid in the target peptide; 4) the amino acid composition of the target peptide; and 5) the composition of adjacent amino acid pairs in the peptide (see supplementary materials for details). These auxiliary tasks serve as the guidance and regulations for the de novo sequencing task. Rather unexpectedly, predicting the length of the target peptide could significantly improve the training stability during the early stages. Additionally, predicting amino acid occurrences (rather than their exact locations) serves as a simple but beneficial task that guides the model throughout its preliminary stages, which reached approximately 90% training accuracy only after the first epoch. Contrastively, the prediction of the existence of adjacent amino-acid pairs shows its value during the final stages. It is the only task that remains improving at the last few epochs of training (although only marginally), thus potentially contributing to the final refinement of the model. The ablation study of the auxiliary tasks shows that, while the impact of each individual task appears negligible, their combined effect yields a modest yet consistent improvement (around 0.006 improvement in positional accuracy compared to the baseline, as shown in Supplementary Fig. S10). Therefore, we conclude that incorporating auxiliary tasks during training is beneficial, especially considering that these auxiliary tasks consume less than 1% FLOPS (floating point operations per second) within the model.

### Training datasets and process

To compile the training data set, we collected HCD spectra from multiple peptide spectral libraries including the NIST HCD library^39^, the NIST Synthetic HCD library^39^, the Human HCD library from MassIVE^40^, and the synthetic HCD library from ProteomeTools^13^. The number of spectra in these libraries is summarized in Supplementary Table S1. In total, by retaining spectra with the charges of 1+ to 8+ and from peptides of length no longer than 30, we collected 3,041,570 HCD-MS/MS spectra from 1,066,296 distinct peptides.

The whole data set was randomly split into the training set containing 2,908,323 (95.6%) spectra, the cross-validation set containing 54,018 (1.8%) spectra, and the testing set which contained the remaining 79,229 (2.5%) spectra. Note that this testing set is only used to evaluate the training process, while all results in this paper were reported on other two independent large-scale proteomics datasets. We ensured that the training set, the cross-validation set, and the testing set shared no spectra from the same peptide in order to avoid information leakage.

We use the RAdam optimizer^32^ with the learning rate of 0.02 to train the model for 50 epochs (8 GPUs, batch size of 32 spectra per GPU). The complete training process takes around 80 hours using 8 cards of NVIDIA A6000 GPU. We pick the model weight from the epoch that performs best on the cross-validation set after the training is completed. More details can be found in the “Training process" section of the supplementary materials.

### Configuration of PointNovo and DeepNovo

To build PointNovo and DeepNovo for comparison, we directly used the source codes provided by their original publication. Both models were retrained using the same training set employed by PepNet. As shown in the Supplementary section “Training PointNovo and DeepNovo", we observed no significant performance improvement under various combinations of key hyperparameters (e.g., the learning rate, the dropout rate, and the batch size). Therefore, we choose the weights trained using the unaltered hyper-parameters as specified in their original source codes. Both models converge within predefined training epochs and the best weight on the validation set is selected for testing. Both PointNovo and DeepNovo were executed using their default configurations without modifications. For DeepNovo-DIA, we directly used the pre-trained weights provided by the original publication.

### Supplementary information

Supplementary results

### Source data

Source Data

## Data Availability

The proteomic data used for this study were taken from previous datasets with ProteomeXchange identifiers of PXD019483 [https://proteomecentral.proteomexchange.org/PXD019483] and PXD014877 [https://proteomecentral.proteomexchange.org/PXD014877], the MaxQuant search results provided in these studies were directly reused. The trained models of PepNet have been deposited in the Zenodo database under accession code 7869847 [https://zenodo.org/record/7869847]. The experiment results of PepNet have been deposited in the Zenodo database under accession code 7869927 [https://zenodo.org/record/7869927]. Source data are provided with this paper.

## Code availability

Source code and scripts are available on GitHub at https://github.com/lkytal/pepnet^41^.
