# Supplementary material for: Accurate de novo peptide sequencing using fully convolutional neural networks
Source: Nat Commun. 2023 Dec 2;14:7974. doi: 10.1038/s41467-023-43010-x (PMC10693636; doi:10.1038/s41467-023-43010-x)
Supplement: Supplementary file 1 — Supplementary results [file 41467_2023_43010_MOESM1_ESM.pdf]

Supporting Information

Accurate *De Novo* Peptide Sequencing Using Fully Convolutional Neural Networks

Kaiyuan Liu[1], Yuzhen Ye[1], Sujun Li[1,2], and Haixu Tang[1]

[1]Luddy School of Informatics, Computing, and Engineering, Indiana University, Bloomington, Indiana 47405, United States
[2]Dengding BioAI Co., Ltd., United States

Contents

1 Supplementary Note 1: Training data S-2

2 Supplementary Note 2: Matrix representation of the model output S-2

3 Supplementary Note 3: Numbers of testing spectra from each organism in the dataset PXD014877 S-3

4 Supplementary Note 4: Sequencing results on selected organisms S-3

5 Supplementary Note 5: Sequencing results on unidentified spectra of PXD019483 S-4

6 Supplementary Note 6: Observed factors related to performance S-5

7 Supplementary Note 7: Orthogonal measurements validation S-5

8 Supplementary Note 8: *De novo* sequencing results on the DIA spectra. S-6

9 Supplementary Note 9: Training process S-7

9.1 Details about the auxiliary tasks S-7
9.2 Ablation study of the auxiliary tasks S-7

10 Supplementary Note 10: Optimizing Hyper-parameters for PepNet's Main Architecture S-8

11 Supplementary Note 11: Training PointNovo and DeepNovo S-8

1 Supplementary Note 1: Training data

As mentioned in the article, for training purposes, the HCD spectra were collected from multiple peptide spectral libraries including the NIST HCD library (1), the NIST Synthetic HCD library (1), the Human HCD library from MassIVE (2), and the synthetic HCD library from ProteomeTools (3). The numbers of spectra in these libraries are summarized in Table S1.

Table S1: Numbers of curated HCD spectra and unique peptides in the spectral libraries that are used for the training, cross-validation, and testing for PepNet. We note that there are no spectra of the same peptides shared by the training, cross-validation, and testing sets.

	Training set size	Unique training peptides	Validation set size	Unique validation peptides	Testing set size	Unique testing peptides
NIST HCD	891,980	288,532	25,282	5,202	35,193	7,446
NIST Synthetic	554,755	161,378	8,441	2,371	12,854	3,514
MassIVE	1,207,959	880,993	16,689	10,195	25,689	15,668
ProteomeTools	253,629	194,687	3,606	2,700	5,493	4,049

2 Supplementary Note 2: Matrix representation of the model output

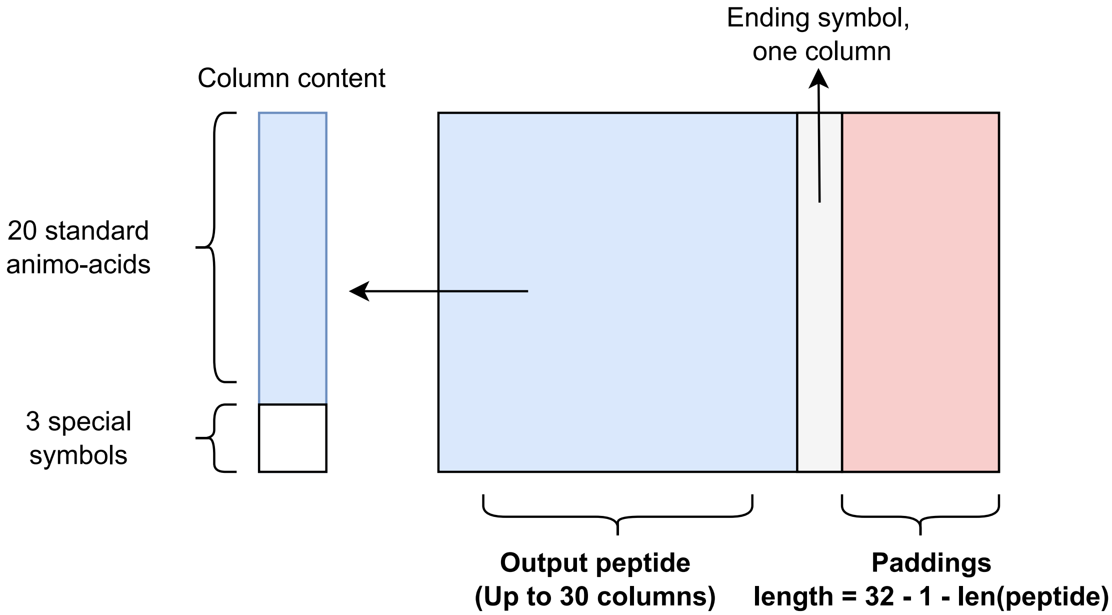

Figure S1: The details about the matrix representation of the model output.

3 Supplementary Note 3: Numbers of testing spectra from each organism in the dataset PXD014877

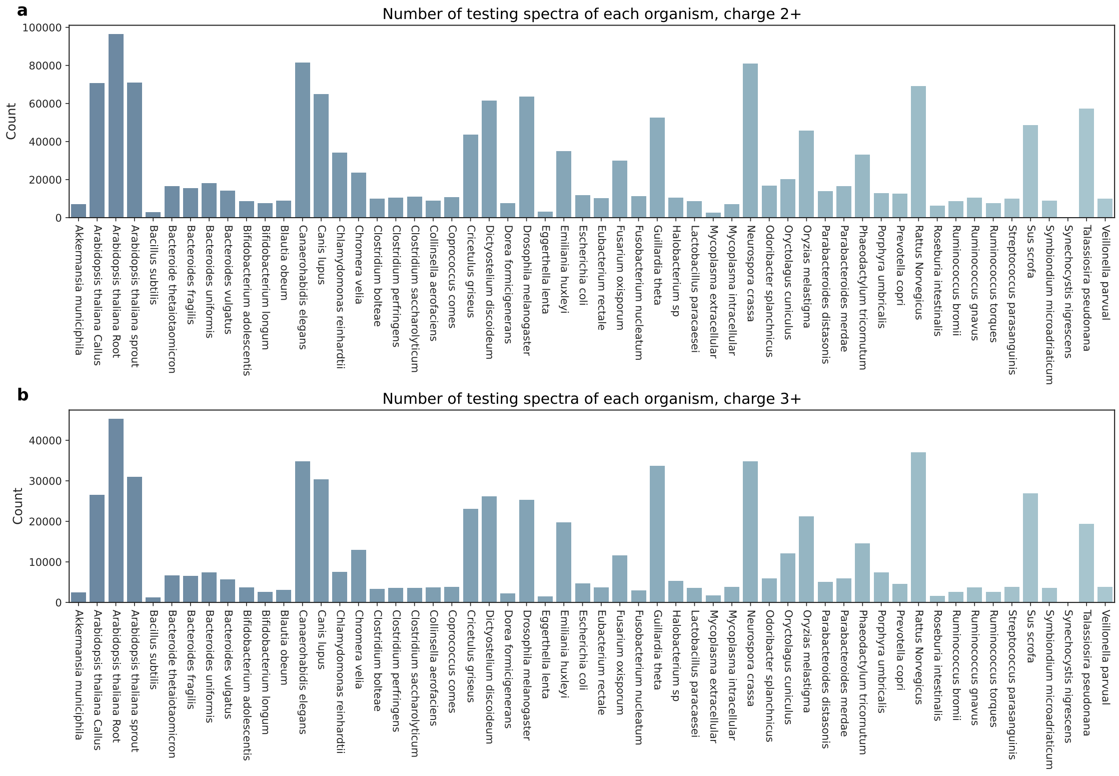

Figure S2: The numbers of testing spectra of charge 2+ (a) and charge 3+ (b) from each organism. Source data are provided as a Source Data file.

4 Supplementary Note 4: Sequencing results on selected organisms

For the two randomly selected organisms ("Arabidopsis thaliana Callus" and "Sus scrofa", respectively). We use PepNet to sequence all spectra (including both identified and unidentified spectra by MaxQuant) obtained from these two organisms in the proteomics study of PXD014877, and then search the sequenced peptides against the corresponding Uniprot database using RAPSearch2. Also, we applied the cutoff scores that can yield 95% peptide-level accuracy based on the identified spectra by MaxQuant from the proteomics data of the corresponding organisms. For Arabidopsis thaliana Callus, among 92,572 sequenced spectra above the score cutoff and with a precursor mass difference no greater than 10 ppm, 39,943 (43.1%) and 3,742 (4.04%) peptides matched with proteins from the organism in the Uniprot database with no or one mutation, respectively. For Sus scrofa, given the number of reviewed proteins in Uniprot is insufficient, we expanded the protein database by incorporating proteins in UniProt unreviewed (TrEMBL). The result showed that, among 107,509 spectra sequenced by PepNet above the cutoff and with s precursor mass difference no greater than 10 ppm, 58,362 (54.3%) and 4,252 (3.96%) matched proteins in the database with no or one mutation, respectively.

5 Supplementary Note 5: Sequencing results on unidentified spectra of PXD019483

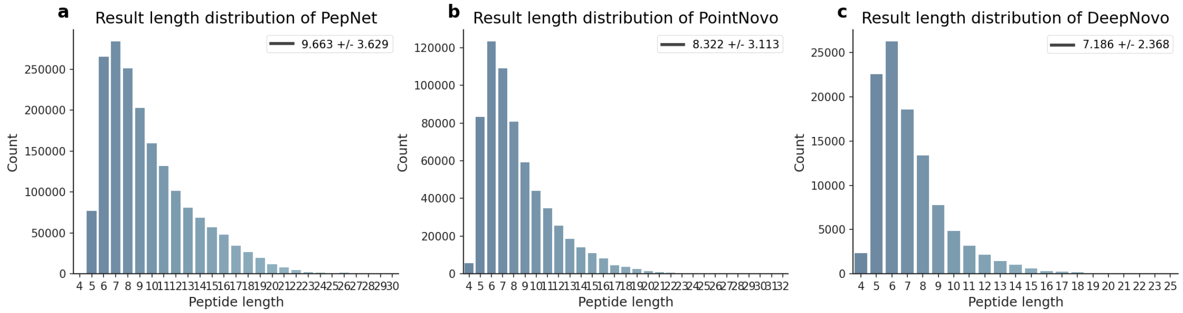

Figure S3: The length distribution of the peptides sequenced by PepNet (a), PointNovo (b), and DeepNovo (c) on the unidentified spectra, after applying the quality score cutoffs. Source data are provided as a Source Data file.

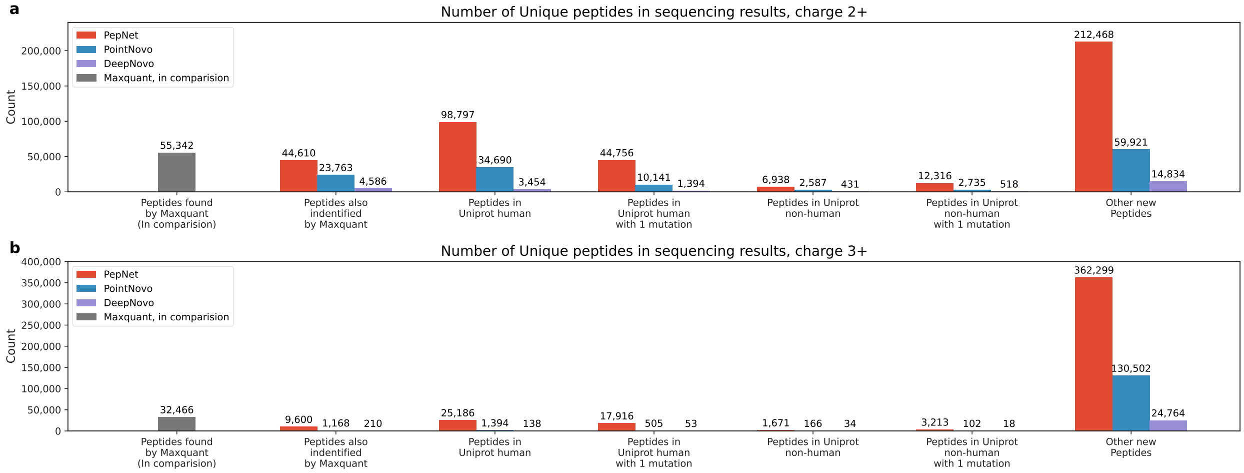

Figure S4: The comparison of unique peptides sequenced by PepNet, PointNovo, and DeepNovo on the unidentified spectra and their matches with the proteins in Uniprot, where the identical matches and the matches with one mutation are plotted separately. (a) Charge 2+ spectra, and (b) charge 3+ spectra. Source data are provided as a Source Data file.

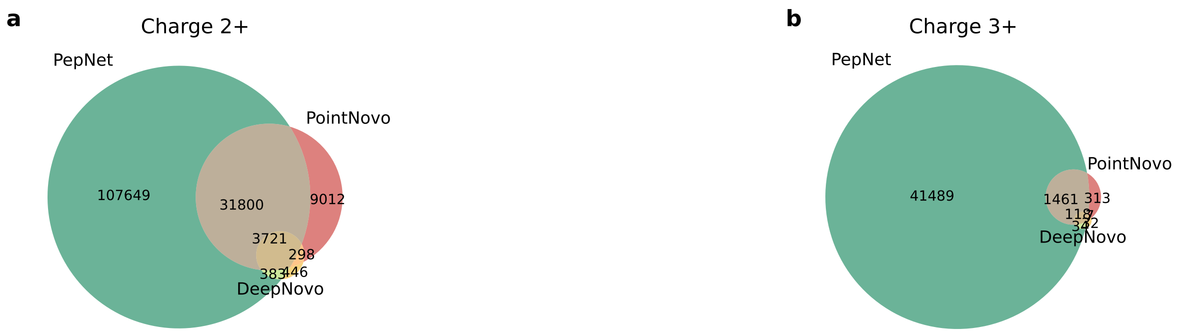

Figure S5: Venn diagram among the peptides sequenced by PepNet, DeepNovo, and PointNovo that match with human proteins in Uniprot for (a) charge 2+ spectra and (b) charge 3+ spectra. Source data are provided as a Source Data file.

6 Supplementary Note 6: Observed factors related to performance

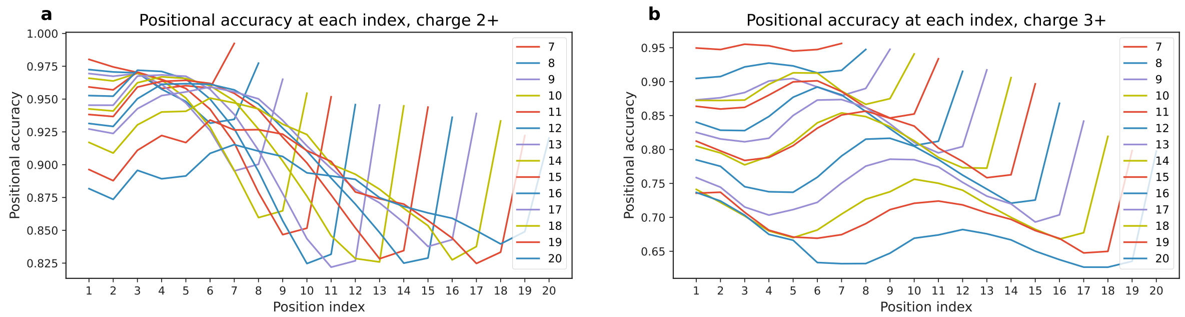

Figure S6: The positional accuracy at each index of the peptides, for sequenced peptide length from 6 to 20. (a) For 2+ spectra, (b) for 3+ spectra. Source data are provided as a Source Data file.

7 Supplementary Note 7: Orthogonal measurements validation

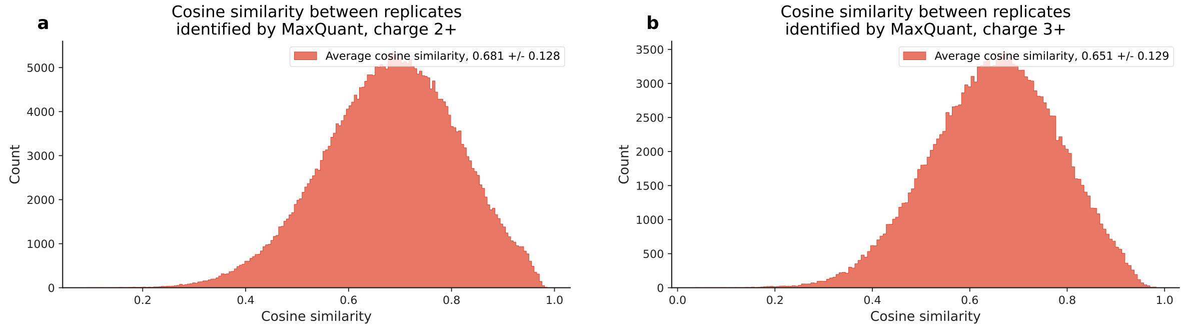

Figure S7: The similarity distributions between replicates (spectra identified with a same peptide) for spectra identified by MaxQuant for (a) charge 2+ spectra, and (b) charge 3+ spectra. Source data are provided as a Source Data file.

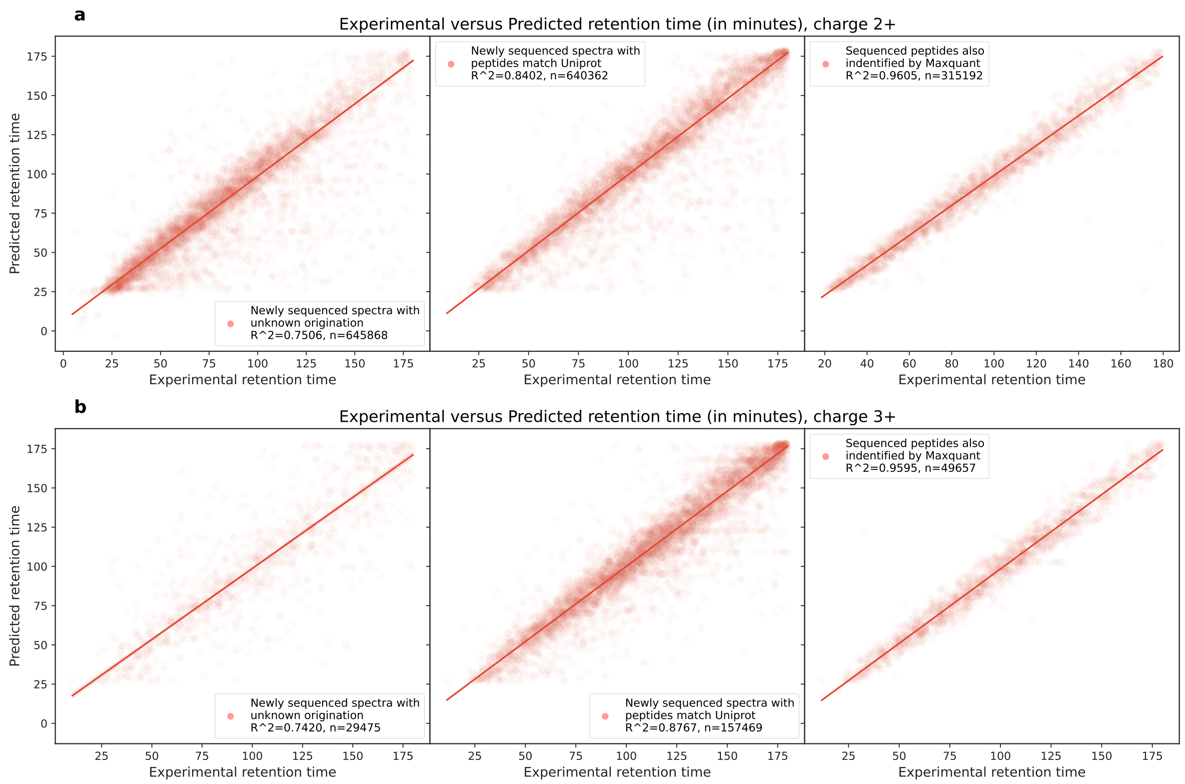

Figure S8: The experimental versus predicted (by DeepLC(4)) retention time on the charge 2+ (a) and charge 3+ (b) spectra (in minutes). Source data are provided as a Source Data file.

8 Supplementary Note 8: *De novo* sequencing results on the DIA spectra.

The *de novo* sequencing results are plotted separately for the DIA spectra of charges 2+ and 3+.

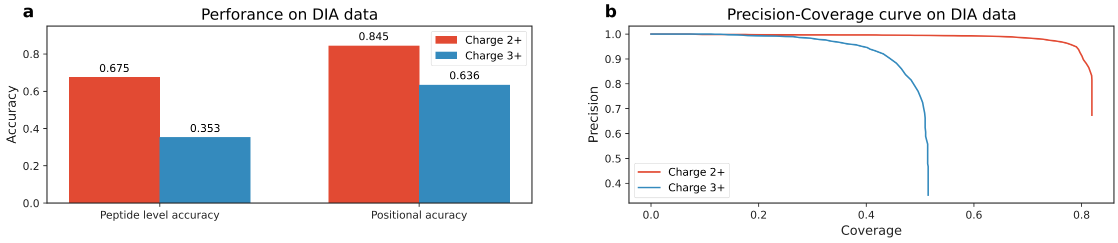

Figure S9: The positional and peptide-level accuracy (a) and the Precision-Coverage curves (b) on the DIA spectra of 2+ and 3+ charges. Source data are provided as a Source Data file.

9 Supplementary Note 9: Training process

For a large model like PepNet, we choose a relatively high learning rate of 0.02 (chosen based on experiments), and use the RAdam optimizer that could simulate a warm-up stage in early training epochs. We first train our model for at most 40 epochs and pick the best set of weights according to the model's performance on the validation set. We notice that in most cases, the model stopped improving after around 30 epochs. After that, we further fine-tune the model for 10 extra epochs using the Adam optimizer with a learning rate of 0.0006.

The impact of the batch size is minor; we set it to 256 (on 8 GPUs, 32 spectra per GPU) to balance the speed and the memory consumption.

9.1 Details about the auxiliary tasks

The auxiliary tasks used for training PepNet and their loss function include:

- whether the target peptide is a tryptic peptide, for which the binary cross entropy is used as the loss;
- the length of the target peptide, for which the loss function is set to be the sum of the categorical cross entropy and the mean square error (MSE);
- the amino acid composition of the target peptide, for which the sparse categorical cross entropy is used as the loss;
- the composition of adjacent amino acid pairs in the peptide, for which the binary cross entropy is used as the loss;
- the existence of 20 amino acids, for which the binary cross entropy is used as the loss for each amino acid;

The weights for the auxiliary tasks are relatively small (as they should not overtake the main task), and are chosen based on the experiments (see the source code for details).

9.2 Ablation study of the auxiliary tasks

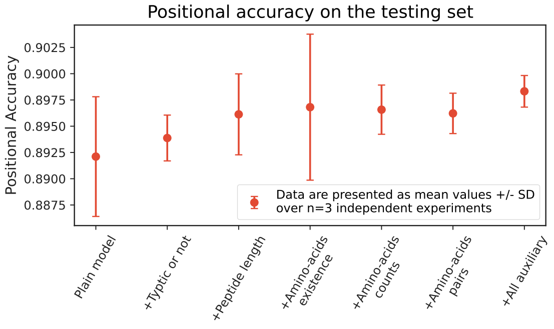

Figure S10: Positional accuracy on the testing set, for the plain model and models with auxiliary tasks. The error bar represents the standard deviation over n=3 repeated training. Source data are provided as a Source Data file.

10 Supplementary Note 10: Optimizing Hyper-parameters for PepNet's Main Architecture

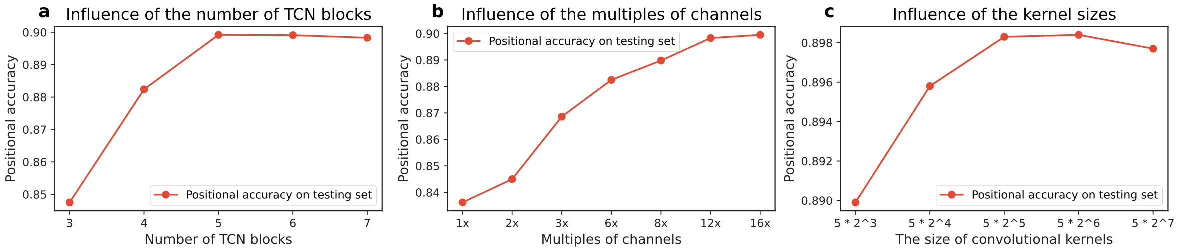

Figure S11: The impact of hyper-parameters on the training performance, for (a) the number of TCN blocks, (b) the number of channels, and (c) the size of convolutional kernels. Source data are provided as a Source Data file.

11 Supplementary Note 11: Training PointNovo and DeepNovo

We retrained both models using the same training set as PepNet.

Here we present the training loss under different combinations of several important hyperparameters (e.g., the learning rate, the dropout rate, and the batch size). As shown in the figure below, we observed no significant performance improvement; thus, the model weights trained using the original hyper-parameters were chosen in our comparison.

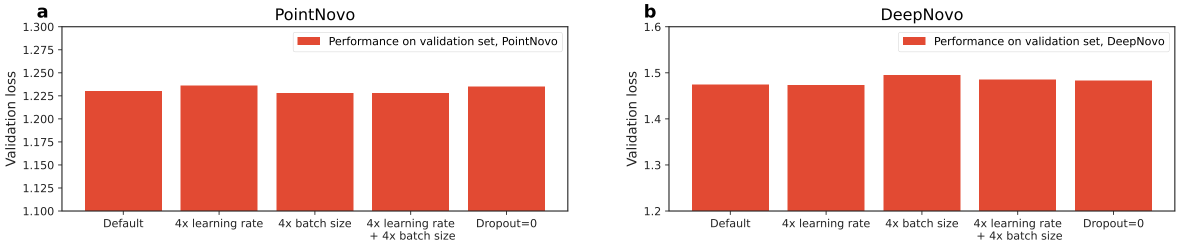

Figure S12: Training loss under different combinations hyperparameters for PointNovo (a) and DeepNovo (b). Source data are provided as a Source Data file.

References

[1] Yang, X., Neta, P. & Stein, S. E. Extending a tandem mass spectral library to include ms 2 spectra of fragment ions produced in-source and ms n spectra. *Journal of The American Society for Mass Spectrometry* **28**, 2280–2287 (2017).

[2] Wang, M. *et al.* Assembling the community-scale discoverable human proteome. *Cell systems* **7**, 412–421 (2018).

[3] Zolg, D. P. *et al.* Building proteometools based on a complete synthetic human proteome. *Nature methods* **14**, 259 (2017).

[4] Bouwmeester, R., Gabriels, R., Hulstaert, N., Martens, L. & Degroeve, S. Deeplc can predict retention times for peptides that carry as-yet unseen modifications. *Nature methods* **18**, 1363–1369 (2021).
